# Impact of genomic profiling on the treatment and outcomes of patients with advanced gastrointestinal malignancies

**DOI:** 10.1002/cam4.992

**Published:** 2016-12-28

**Authors:** Mashaal Dhir, Haroon A. Choudry, Matthew P. Holtzman, James F. Pingpank, Steven A. Ahrendt, Amer H. Zureikat, Melissa E. Hogg, David L. Bartlett, Herbert J. Zeh, Aatur D. Singhi, Nathan Bahary

**Affiliations:** ^1^ Division of Surgical OncologyUniversity of Pittsburgh Medical CenterPittsburghPennsylvania15232; ^2^ Division of Gastrointestinal PathologyUniversity of Pittsburgh Medical CenterPittsburghPennsylvania15232; ^3^ Division of OncologyUniversity of Pittsburgh Medical CenterPittsburghPennsylvania15232

**Keywords:** Clinical benefit, gastrointestinal malignancies, genomic‐guided therapy, genomics, precision medicine

## Abstract

The impact of genomic profiling on the outcomes of patients with advanced gastrointestinal (GI) malignancies remains unknown. The primary objectives of the study were to investigate the clinical benefit of genomic‐guided therapy, defined as complete response (CR), partial response (PR), or stable disease (SD) at 3 months, and its impact on progression‐free survival (PFS) in patients with advanced GI malignancies. Clinical and genomic data of all consecutive GI tumor samples from April, 2013 to April, 2016 sequenced by FoundationOne were obtained and analyzed. A total of 101 samples from 97 patients were analyzed. Ninety‐eight samples from 95 patients could be amplified making this approach feasible in 97% of the samples. After removing duplicates, 95 samples from 95 patients were included in the further analysis. Median time from specimen collection to reporting was 11 days. Genomic alteration‐guided treatment recommendations were considered new and clinically relevant in 38% (36/95) of the patients. Rapid decline in functional status was noted in 25% (9/36) of these patients who could therefore not receive genomic‐guided therapy. Genomic‐guided therapy was utilized in 13 patients (13.7%) and 7 patients (7.4%) experienced clinical benefit (6 PR and 1 SD). Among these seven patients, median PFS was 10 months with some ongoing durable responses. Genomic profiling‐guided therapy can lead to clinical benefit in a subset of patients with advanced GI malignancies. Attempting genomic profiling earlier in the course of treatment prior to functional decline may allow more patients to benefit from these therapies.

## Introduction

Genomics‐driven cancer treatments have provided new hope to cancer patients with advanced malignancies. This approach is often termed as precision oncology or personalized cancer care 1, 2. Although oncologists have been customizing the treatment of various malignancies based on site, stage, and tolerability, etc., genetic guidance in selection of therapies has added another level of complexity. Data from retrospective analyses and early‐phase clinical trials have demonstrated that strategy of matching targeted agents with genomic alterations is associated with encouraging results in the treatment of patients with various cancers 3, 4, 5, 6. Schwaederle et al. in their meta‐analysis of phase II clinical trials noted that, across various malignancies, a personalized strategy was an independent predictor of better outcomes and fewer toxicity‐related deaths 6.

However, integration of genomics‐driven care into routine oncology practice remains challenging given the practical and cost implications 1, 2, 7. At the time of their genomic evaluation, many of these patients have advanced refractory disease and a rapidly declining clinical course, disqualifying them from participating in molecular target‐driven clinical trials. There are few prospective studies validating the feasibility of a genomics‐driven approach in solid tumors including single‐disease and multidisease settings 8, 9. Sohal et al. in their prospective study of solid tumors reported that 49% of the 223 evaluable patients were recommended a specific therapy, but only 11% received such therapy 9. However, data evaluating genomics‐driven therapy specifically for advanced gastrointestinal (GI) malignancies are limited. In addition, it remains unknown whether such therapy would impact outcomes as mutations detected late in the treatment course of the disease may not be the current disease drivers. Therefore, the percentage of patients with advanced GI malignancies whose treatment plan is altered after genomic testing remains unknown. Consequently, the aim of this study was to evaluate the impact of next‐generation sequencing on the treatment plan and outcomes in patients with advanced gastrointestinal malignancies.

## Patients and Methods

The study was approved by the University of Pittsburgh Institutional Review Board (PRO16020064). All patients with advanced gastrointestinal malignancies treated at University of Pittsburgh Cancer Institute and allied cancer centers who underwent next‐generation sequencing (NGS) were included in this study. The unique setup of the University of Pittsburgh Medical Center—Cancer Centers Network connects the main institution with >40 network community sites covering a geographic area of >200 miles around greater Pittsburgh allowing us to include patients treated at both academic and community oncology centers.

Tumor specimens were shipped to Foundation Medicine, Inc. (Cambridge, MA), for sequencing using the FoundationOne platform. This platform is designed to include all genes known to be somatically altered in human solid tumors that are validated targets for therapy, either approved or in clinical trials, and/or that are unambiguous drivers of oncogenesis based on current knowledge 10. Technical specifications for the test are provided at the FoundationOne website (http://foundationone.com/learn.php#4). Briefly, FoundationOne applied next‐generation sequencing to identify all four types [base substitutions, insertions and deletions (indels), copy number alterations (CNAs), and rearrangements] of genomic alterations across the coding region of 315 cancer‐related genes plus introns from 28 genes. Funding for testing was borne as standard of care through each individual's insurance plan.

Genomic data were collected from the standard FoundationOne patient reports. Alterations which have been previously characterized in the literature were categorized as gene alterations (GAs), whereas the alterations which have not been characterized in the literature were referred to as variants of unknown significance (VUS). VUSs were not considered to make any therapeutic decisions in this study. Electronic chart review was performed to evaluate for demographic variables of the patients as well as to ascertain if the genomics‐guided therapy was utilized in the treatment of the patients. Statistical analyses were performed using STATA 14.1 (Statacorp, 4905 Lakeway Drive, College Station, Texas, US). Continuous variables were summarized as medians with interquartile range (IQR) and categorical variables were summarized as frequencies and percentages. Progression‐free survival was calculated from the start of genomic‐guided therapy until evidence of disease progression by imaging or tumor markers. Kaplan–Meier method was used to estimate the probability of progression‐free survival. Clinical benefit was defined as complete response (CR) or partial response (PR) or stable disease (SD) at 3 months.

## Results

A total of 101 consecutive samples from 97 patients consisting of all samples sent from the University of Pittsburgh Medical Center—Cancer Centers Network to FoundationOne from April, 2013 to April, 2016 were analyzed. Ninety‐eight samples from 95 patients could be amplified to provide meaningful genetic information making this approach feasible in 97% of the samples. Three patients had two samples each sent for testing; only one sample was included in the analysis to avoid duplication of the genetic information. Therefore, 95 samples from 95 patients were included in the further analysis.

The median age of the studied patients was 50 years; women comprised 40% of patients. Table 1 summarizes the baseline characteristics of the patients and tested samples. Colorectal adenocarcinoma (36%), pancreatic adenocarcinoma (20%), and appendiceal adenocarcinoma (9.5%) were the most common diagnoses. Approximately 28% of the reports were qualified, that is, genomic alterations could be confirmed, but the data obtained may have been insufficient for comprehensive detection of genomic alterations due to poor specimen quality. Median time from specimen receipt to reporting of the genomic data was 11 days and 75% of the samples were reported within 2 weeks. Median number of GAs (excluding VUS) detected per sample were 4 and median number of VUS per sample were 7. Median time from the original diagnosis to genomic profiling was 20 months. A median of 1 FDA‐approved therapy in other tumor types which could be implicated in the tumor being sequenced was suggested. A median of six clinical trials, mainly phase I trials, based on GAs were suggested.

**Table 1:** Baseline characteristics of the patients/samples *N* = 95

	*N* (%)/median (iqr)
Age, year	50 (39–61)
Females	38 (40)
Diagnosis	
Pancreatic Adenocarcinoma	19 (20)
Colorectal Adenocarcinoma	34 (35.8)
Appendiceal Adenocarcinoma	9 (9.5)
NET—pancreas/small bowel	6 (6.3)
Unknown primary adenocarcinoma including PB/UGI type	5 (5.3)
Cholangiocarcinoma	5 (5.3)
Unknown primary—neuroendocrine carcinoma	3 (3.1)
Other	14 (14.7)
Time from specimen collection to receipt, days	0
Time from receipt to reporting, days	11 (9–13)
Time since cancer diagnosis, months	20 (10–39)
Qualified reports	27 (28.4)
Gene Alterations per sample	4 (2–6)
VUS per sample	7 (5–11)
Number of FDA‐approved therapies in the tested tumor type suggested based on genetic testing	0 (0)
Number of FDA‐approved therapies in other tumor types implicated in treatment	1 (0–3)
Number of suggested clinical trials	6 (3–10)
Prediction of lack of response to standard therapies	15 (16)

NET, Neuroendocrine tumor; PB, pancreaticobiliary; UGI, Upper gastrointestinal; VUS, Variants of unknown significance; FDA, Food and drug administration.

**Figure 1:**
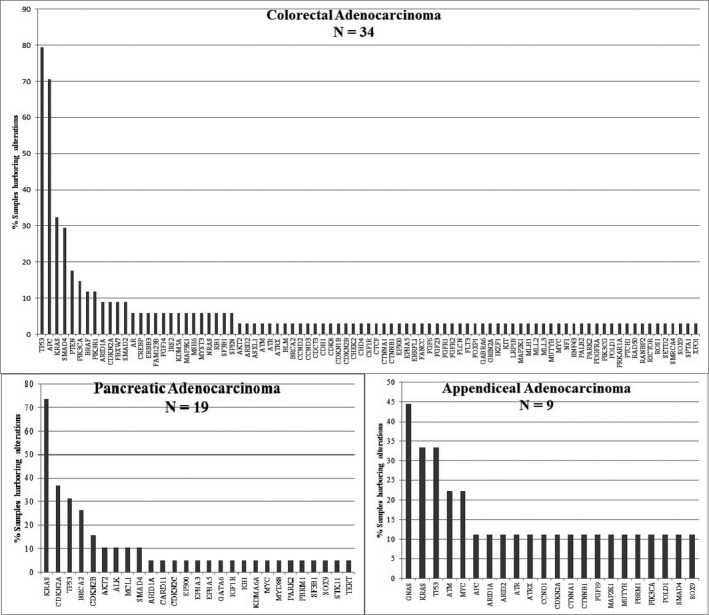
Mutation frequencies of genes in colorectal, pancreatic, and appendiceal adenocarcinoma.

TP53, APC, KRAS, and SMAD4 were the most frequently mutated genes in colorectal adenocarcinoma. KRAS, CDKN2A, and TP53 were frequently mutated in pancreatic adenocarcinoma. Interestingly, 25% of tumors from patients with pancreatic adenocarcinoma harbored a BRCA2 mutation. GNAS, KRAS, TP53, and ATM were frequently mutated in appendiceal adenocarcinoma tumors. Figure 1 provides a summary of genetic alterations discovered in the three most common tumor types, that is, colorectal adenocarcinoma, pancreatic adenocarcinoma, and appendiceal adenocarcinoma.

**Figure 2:**
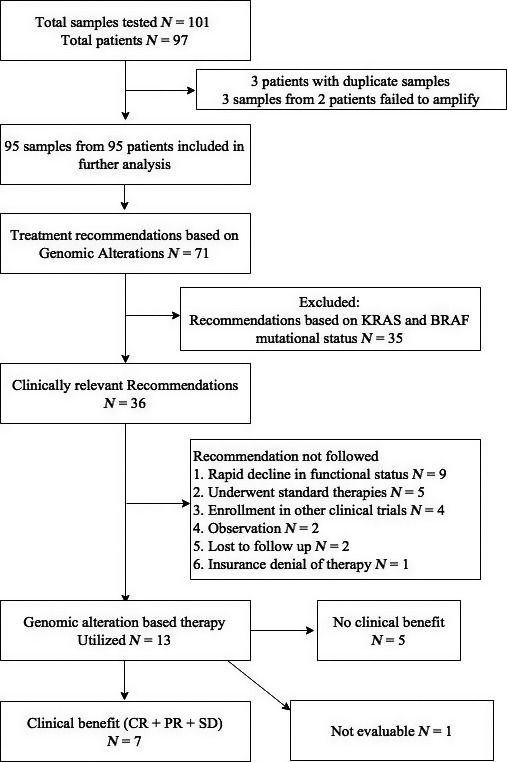
Flow diagram providing a summary of samples.

Treatment recommendations based on GAs were suggested for 75% (71/95) of the patients (Fig. 2). However, in our institution, KRAS and BRAF mutations were not considered clinically relevant as most patients with CRC already had these tests performed as a matter of protocol, and no directed KRAS therapies exist. Therefore, recommendations were considered new and clinically relevant in 38% (36/95) of the patients. In approximately two thirds of these patients (23/36), genomic‐guided therapy was not instituted due to several reasons including: (1) rapid functional decline in the patient (*N* = 9), (2) use of alternative standard of care therapies (*N* = 5), (3) enrollment in other clinical trials (*N* = 4), (4) observation/no evidence of disease (NED) after only site of disease was resected (*N* = 2), (5) lost to follow‐up (*N* = 2) and (6) insurance denial of therapy (*N* = 1). Genomic‐driven therapy was utilized in 13 patients (13.7%, 13/95). A summary of these patients is provided in Table S1. Only one patient received such therapy under a clinical trial (described below).

**Figure 3:**
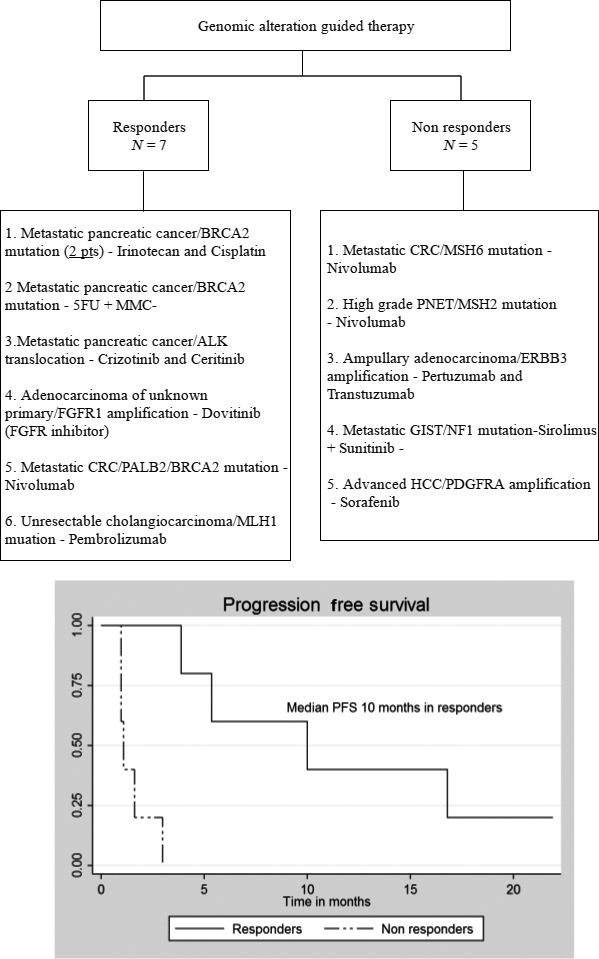
Summary of patients treated with genomic‐guided therapy and progression‐free survival. One patient with BRCA2 mutation and metastatic pancreatic cancer recently started irinotecan and cisplatin and was not evaluable.

Many of the patients who responded to treatment based on a GA had alterations in the DNA damage repair pathway. Four patients with metastatic pancreatic cancer were found to have BRCA2 mutations. Two of these patients had a partial radiologic response and normalization of their CA 19‐9 in response to irinotecan and cisplatin. A third patient in this group received 5‐Fluorouracil and mitomycin C–based therapy due to chronic renal insufficiency and also had an excellent partial radiological response with necrosis in areas of disease and CA 19‐9 normalization. A fourth patient with a BRCA2 mutation and metastatic pancreatic cancer only recently started platinum‐based therapy and was not evaluable. Given limited open regional protocols, we have reserved therapy with PARP inhibitors for a later time point. A 30‐year‐old woman with a past history of treated early‐stage colon cancer at age 17 presented with recurrent metastatic colon cancer. Tumor analysis revealed a hypermutator phenotype; her tumor harbored more than 600 mutations/Megabase (Mb). Further evaluation of her tumor failed to reveal an underlying mismatch repair (MMR), DNA excision repair, and POLE or POLD mutations. Having failed all standard previous therapies (multiple surgeries including left colectomies, three cytoreductive procedures; two of which were accompanied with intraperitoneal hyperthermic chemoperfusion with mitomycin C, FOLFOX, FOLFIRI, and FOLFIRINOX with cetuximab) she was approved for, and responded to the anti‐PD‐1 agent nivolumab 11. Three patients with mutations in the MMR genes were treated with PD‐1 inhibitors: (1) one patients with unresectable cholangiocarcinoma who had an MLH1 mutation had a near‐complete and persistent response to pembrolizumab (discussed below), and (2) another heavily pretreated patient's metastatic CRC tumor had an MSH6 mutation and another patient's metastatic well‐differentiated high‐grade pancreatic neuroendocrine tumor harbored an MSH2 mutation (this patient's microsatellite status could not be confirmed by IHC). However, as they had exhausted all standard treatments options (mCRC KRAS mut—FOLFOX and bevacizumab, NET—interferon, long‐acting octreotide analogues, everolimus, peptide receptor radiotherapy ×2, multiple chemoembolizations, combination of everolimus and bevacizumab, temozolamide and capecitabine, XELOX, sunitinib alone, and in combination with hydroxychloroquine) they were treated with, but failed to respond to Nivolumab and ultimately succumbed to their disease. Interestingly, one patient with metastatic pancreatic cancer was found to have an ALK translocation and responded to ALK inhibition. Additionally, one patient with metastatic adenocarcinoma of unknown primary was found to have FGFR amplification and was treated with the FGFR inhibitor dovitinib on a clinical trial, resulting in stabilization of disease. In addition to the two patients with defects in mismatch repair genes (MHS2 and MSH6) who did not respond to nivolumab, three patients with therapies guided by ERBB3 amplification, NF1 mutation, and PDGFRA amplification failed to respond to directed therapy. Figure 3 provides summary of all the study patients. Median progression‐free survival (PFS) was approximately 4 months in all patients who received genomic‐guided therapy and was 10 months among those who derived any clinical benefit. A description of three patients who derived benefit from genomic‐guided therapy is provided below. These three patients were chosen as they had both a biochemical and radiologic response for illustration.

### Case 1—BRCA2 mutation and use of irinotecan with cisplatin in metastatic pancreatic cancer

**Figure 4:**
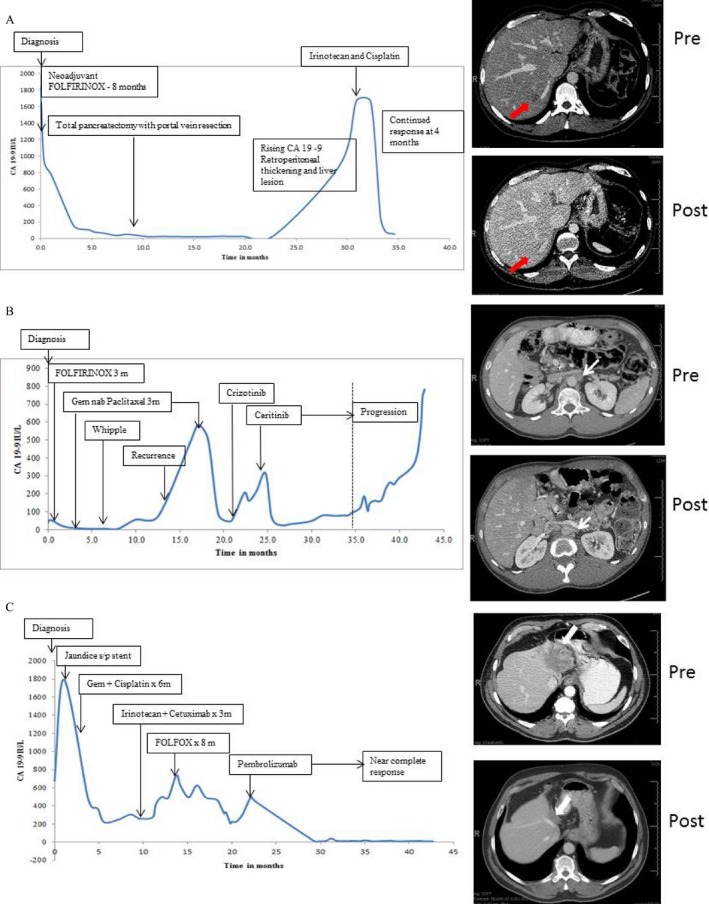
(A) CA 19‐9 and radiologic response in a patient with metastatic pancreatic cancer found to have BRCA2 mutation and treated with irinotecan and cisplatin; (B) CA 19‐9 and radiologic response in a patient with metastatic pancreatic cancer treated with ALK inhibitors crizotinib and ceritinib; (C) CA 19‐9 and radiologic response in a patient with unresectable cholangiocarcinoma treated with pembrolizumab. Pre and Post indicate pretherapy and posttherapy scans. Arrows mark the index lesions that were most easy to demonstrate and compare on the scans.

The patient was a 38‐year‐old man, with no family history of cancer, initially diagnosed with biopsy‐proven locally advanced unresectable pancreatic (head/body) adenocarcinoma in 2013. He was treated with 8 months of FOLFIRINOX (5‐Fluorouracil, Leucovorin, Irinotecan, and Oxaliplatin) therapy and had a partial response to therapy. He subsequently underwent total pancreatectomy with portal vein resection with negative margins. Final pathology revealed a 6.5‐cm tumor, ypT3N1, and 13/47 lymph nodes positive for adenocarcinoma. No adjuvant therapy was given due to decline in functional status at that time. Eighteen months postresection, an elevation in CA 19‐9 was noted along with soft tissue thickening around the celiac axis, gastrohepatic ligament, subtle hypodensity in the segment 7 of the liver, and omental nodularity suggestive of early carcinomatosis. An endoscopic ultrasound‐guided biopsy of the celiac region was positive for adenocarcinoma and this specimen was submitted for genomic analysis. Genomic analysis identified biallelic BRCA2 mutation along with KRAS, SMAD4, and CDKN2A mutations. He was started on irinotecan and cisplatin. Pretherapy CA 19‐9 was 1673 IU/L and after four cycles CA 19‐9 decreased to 52.9 IU/L (Fig. 4A). He is currently continuing on therapy with continued response at 4 months.

### Case 2—ALK translocation and use of ceritinib in metastatic pancreatic cancer

This patient was a 35‐year‐old man diagnosed with biopsy‐proven locally advanced pancreatic head adenocarcinoma in 2012 and underwent 3 months of therapy with FOLFIRINOX. Therapy was subsequently switched to gemcitabine and nab‐paclitaxel due to lack of response. He underwent another 3 months of gemcitabine and nab‐paclitaxel followed by pancraticoduodenectomy with negative margins. Final pathology revealed a 2‐cm tumor, ypT3N1, and 2/56 lymph nodes positive for adenocarcinoma. Postoperative adjuvant therapy was deferred due to poor functional and nutritional status. Ten months postresection CA 19‐9 was noted to be 585 U/mL and CT imaging revealed multiple newly enlarged retroperitoneal, periaortic, and retrocaval lymph nodes. At this time gemcitabine and nab‐paclitaxel were reinstituted. His CA 19‐9 normalized, but therapy was discontinued after 3 months secondary to severe neuropathy and fatigue. At that time, tumor from his resection specimen was sent for genomic analysis revealing an ALK‐EML4 translocation. He was started on Crizotinib without significant biochemical response possibly due to multiple needed dose reductions for hematologic toxicity. Treatment was then subsequently changed to Ceritinib leading to normalization of his CA 19‐9 as well as radiographic response and clinical improvement until progression after approximately 6 months of therapy (Fig. 4B). At this time repeat genomic analysis was performed which revealed the same ALK translocation. A recommendation was made for changing to an alternative ALK inhibitor—alectinib and insurance approval is currently pending.

### Case 3—MLHI mutation and use of pembrolizumab in unresectable cholangiocarcinoma

A 60‐year‐old man presented in October 2012 with abdominal pain. A CT scan demonstrated a 4.6‐cm mass in the left liver with extension into the portahepatis and encasement of the common hepatic artery and portal vein; biopsy was consistent with cholangiocarcinoma. Within a month he developed jaundice. His EUS confirmed T4N0 disease and ERCP was performed with placement of a stent. The patient underwent eight cycles of gemcitabine and cisplatin, which was discontinued due to toxicity. A repeat CT scan in June, 2013, revealed stable disease. He then underwent six cycles of irinotecan and cetuximab followed by a CT scan in September 2013, which demonstrated progression of disease. His therapy was changed to FOLFOX, which was discontinued after 13 cycles due to thrombocytopenia and progressive peripheral neuropathy. In July, 2014, NGS/FoundationOne testing identified biallelic MLH1 loss. The patient subsequently enrolled in a clinical trial with pembrolizumab. After approximately 6 months, he had an excellent partial response to therapy and is currently continuing treatment on pembrolizumab (Fig. 4C).

## Discussion

This study highlights the role of genomic profiling using a commercially available platform in patients with advanced GI malignancies being treated in a GI oncology practice including both academic and community cancer centers. Our data suggest that this approach is feasible as 97% of the samples could be successfully amplified and median time to reporting was 11 days. Recommendations for therapy were new and clinically relevant in 38% of the patients, but only 13.7% eventually received genomic‐guided therapy. The majority of these patients had advanced disease and a rapid clinical decline that prevented them from receiving genomic‐guided therapy off‐label or enrollment in an appropriate clinical trial. This suggests that undertaking genomic tumor profiling earlier in a patient's disease course may facilitate the identification and utility of directed protocol therapies. Of those screened in this analysis, however, approximately 7% of the patients eventually experienced clinical benefit. The most common single targetable alteration identified was a previously undetected BRCA2 mutations in pancreatic cancers. Multiple specific chemotherapy regimens can target this mutational landscape and we present responses with both platinum‐ and mitomycin C‐based regimens in BRCA2‐mutated patients. Trials with PARP inhibition in patients with BRCA2 mutations are also underway. Given the prevalence of BRCA pathway mutations in pancreatic adenocarcinoma (PDAC), we believe that BRCA pathway testing should be routinely undertaken in PDAC. The next most common clinically useful alteration was in the pathway of genomic instability; including one patient with metastatic CRC and hypermutator phenotype of unknown source and another patient with unresectable cholangiocarcinoma and MLH1 mutation who is responding to pembrolizumab. However, other unique molecular alterations were also identified that proved useful in offering targeted treatments (Fig. 3).

The potential for molecular‐guided therapy to affect the treatment of an individual patient has been recognized 12, 13. Initial studies by Sjoblom et al. defined the genetic landscapes of breast and colorectal cancer in 2006 and not only described several novel genes but also identified some of the key dysregulated pathways which could be the targets of therapy in future 14. Since then a team approach to defining the mutational landscape of various cancers have been taken by The Cancer Genome Atlas Project and International Cancer Genome Consortium, and new mutations and dysregulated pathways have been defined in several cancers 15, 16. Several authors have previously reported cases where drugable targets were identified and significant improvement in progression‐free survival was observed 5, 17. Others have verified the feasibility of high‐throughput testing of clinical samples for various mutations 18. Boland et al. reported their results of 500 patients who underwent next‐generation sequencing of a panel of 46 genes in a phase I clinical trials program 19. Seventy‐two percent of these patients had a mutation or variant among the 46 tested genes and 30% of the patients had alterations which were potentially actionable 19. Additionally, a recent meta‐analysis of phase II clinical trials suggested that a personalized approach compared to a nonpersonalized approach was associated with an improvement in response rates (31% vs. 10.5%, *P* < 0.001), progression‐free survival (5.9 months vs. 2.7 months, *P* < 0.001), overall survival (13.7 months vs. 8.9 months, *P* < 0.001), and led to fewer toxic deaths. These studies have encouraged many to consider genomics‐guided therapies in patients with advanced malignancies.

Major areas of investigation with molecular‐derived therapy include: (1) feasibility and timeliness of high‐throughput testing, (2) actionability of the targets identified, (3) and most importantly, effect on patient outcomes. Our results are discussed in these three contexts.

### Feasibility and timeliness of reporting

Our results are comparable to others studies which reported a median time of 18–26 days from consent to reporting 8, 9, 20, 21. Of note, as this was a retrospective analysis, time from consent to dispatch of the specimens was not evaluable and this may have led to a shorter median time to reporting of 11 days in this study. Similarly, previous studies have reported feasibility of genomic analyses in 61–86% of the patients 9, 20, 21. In this study only patients who had specimens available for genomic profiling were analyzed and we could not evaluate the number of patients who could not undergo genomic testing due to inadequate specimens. Although molecular profiling could be performed in 97% of the specimens, approximately 30% of the reports were qualified. This could be due to poor specimen quality or sequencing failure, but the exact reasons could not be ascertained.

### “Actionability” of identified targets

The definition of actionability is not uniform and different terms such as “druggable”, “actionable”, “therapy matching”, “genomic guidance”, etc. have been used. Similarly, the impact of a genetic alteration and response to treatment may be different in different tumors, for example, BRAF V600E mutation predicts response to single‐agent vemurafenib in melanoma but not in colorectal cancer 22. Most of the studies have previously reported a targetable/actionable/druggable mutation in 30–49% of the patients 8, 9, 19, 20. In contrast to the study by Boland et al. (discussed above), KRAS mutations were not considered clinically actionable for therapy in this study due to (1) lack of effective agents that target RAS pathway and (2) most patients with metastatic colorectal cancer undergo expanded RAS analysis at our institute prior to Foundations testing 23. Although this study confirmed the presence of previously known mutations in GI tumors, one of the interesting findings was the presence of BRCA2 mutations in 25% of the tested pancreatic adenocarcinoma samples allowing for tailored therapy using platinum‐based or mitomycin‐based agents for these patients. They also support the investigational use of PARP inhibitors in available clinical trials at progression. These results are consistent with the recent reports which suggested a 17–24% incidence of BRCA2/PALB2 mutations in patients with pancreatic adenocarcinoma 24, 25. Additionally, based on the NGS results identifying the MLH1 mutation in one patient with cholangiocarcinoma, as well as other data supporting the incidence of a deficient mismatch repair (dMMR) phenotype in multiple GI cancers, we have routinely started screening all GI tumors for mismatch repair deficiency. Although the true incidence of dMMR in various GI tumors remains uncertain, we believe this is a prudent algorithm given the efficacy of PD‐1 inhibitors in tumors with mismatch repair deficiency 26. As a result, another patient with advanced pancreatic cancer not reported here was found to have an MLH1 gene mutation on our routine screening for MMR genes and treated with a PD1 inhibitor resulting in a partial response.

Efforts for integration of genomic approaches in clinical practice are ongoing. At some institutions panel testing is undertaken for all patients. Other institutions utilize the FoundationsOne or similar platforms. Our own institution is in the process of creating a panel including BRCA and other DNA repair pathway genes. Rather than dictate a single platform we would recommend the platform that is available to the individual oncologist, which will differ whether they are located in the community or at academic institutions.

### Patient outcomes

It remains largely unknown as to what percentage of all patients eventually benefit from genomic‐guided therapy as only a small percentage of mutations are drivers and the vast majority are passenger mutations which confer no growth advantage 27. Additionally, clinical significance of many variants remains unknown at this time. No such variants were used to guide therapy in this study. Several randomized controlled trials are underway to assess the effectiveness of genomic‐guided therapy including IMPACT II (Initiative for Molecular Profiling and Advanced Cancer Therapy II, www.clincaltrials.gov NCT02152254), SHIVA (A Randomized Phase II Trial Comparing Therapy Based on Tumor Molecular Profiling Versus Conventional Therapy in Patients With Refractory Cancer, www.clincaltrials.gov NCT01771458), and NCI‐MPACT (National Cancer Institute—Molecular Profiling‐based Assignment of Clinical Trials, www.clincaltrials.gov NCT01827384)^3^. One of the major barriers to the receipt of genomic‐driven therapy is the rapid functional decline in these patients with advanced malignancies. Most of these clinical trials allow only patients with good functional status which may not be the case in the real world. Sohal et al. in their prospective study of precision oncology in solid tumors reported that 49 (26%) of 109 patients (109/223, 49% of total) who were recommended genomic‐guided therapy could not receive the therapy given rapid functional decline. In this study, 38% of the patients were recommended genomic‐guided therapy, but 25% (9/36) of these patients had rapid functional decline. This raises the question of the timing of genomic profiling with regards to the stage of disease. The median time from diagnosis to genomic profiling in this study was 20 months and was 18 months in the study by Sohal et al. Chantrill et al. in their report of pilot stage of IMPaCT trial identified 22 of 93 patients with potential for receipt of molecular‐guided therapy, but none of these patients were eligible given rapid decline in functional status 8. Earlier testing will allow more patients to have genomic‐guided therapy and possibly positively impact patient outcomes as well.

This study is unique as it focused on advanced GI malignancies and reported the clinical outcomes in patients who received genomic‐guided therapy. Von Hoff et al. in their pilot study reported that 18 (27%) of the 66 patients who received molecular profiling (MP)‐guided therapy demonstrated an improvement in their PFS, that is, PFS on MP‐guided therapy/PFS on most recent therapy >1.3 28. However, only 6 of the 18 (6/86, 7%) patients had GI malignancies. Definitions such as PFS on MP‐guided therapy/PFS on most recent therapy >1.3 may not assess clinical benefit as transparently as disease control at 3 months, as used in this study. Recently, a large single‐center prospective study by Wheler et al. was published wherein the authors reported their experience in 500 patients who underwent NGS using a similar platform as used in this study 29. Approximately 38% (188/500) of the patients received therapy of which 65% (122/500) received matched (genomic‐guided therapy) and 35% (66/188) received unmatched therapy. These authors reported a significant improvement in time to failure in patients who underwent matched therapy (median 2.8 months vs. 1.9 months, *P* = 0.001). Additionally, patients who underwent matched therapy also demonstrated a trend toward improved OS (median 9.2 months vs. 6.8 months, *P* = 0.087) and were more likely to derive clinical benefit (19% vs. 8%, respectively, *P* = 0.061). Similar to this study the most common reasons for nonreceipt of therapy were functional decline/death or hospice transfer. This study only focuses on GI malignancies, whereas in the Wheler et al. study, the majority of the patients had nongastrointestinal malignancies including ovarian (18%), breast (16%), sarcoma (13%), and renal cell cancers (7%).

The limitations of this study include its retrospective design, different tumor types, small sample size of each individual tumor type, and the limited variety of analyzed tumors resulting from inherent local referral patterns and patients' insurance willingness to bear the costs of the analysis. As only a single platform was utilized, underdetection of some targetable mutations in genes recently implicated in genomic instability, such as RPA1, XRCC4, and XRCC6, may have occurred 24, 25. As NGS platforms continue to evolve further actionable/targetable mutations will be analyzed. Additionally, given the small sample size distribution of tumor types in this study, these results may not match the tumors' epidemiologic distribution. Mutant allele frequencies (MAFs), which can provide insight into tumor heterogeneity or germline status, were not available for all patients in this study. Finally, given the possibility of uncovering germline mutations, all efforts should be made to determine if the source of mutations is germline. Genetic counseling should then be sought in patients found to have germline mutations.

In conclusion, current platform genomic profiling results can be obtained in most patients within 2 weeks, and up to one third of the patients may be candidates for genomic‐guided therapy. However, only a small percentage of patients are able to receive such therapies given that most patients had such determinations made late in the clinical course, when they are facing a rapid functional decline. Genomic profiling undertaken earlier in a patient's clinical course may afford an improved chance to undergo directed therapy or afford time to access clinical trials of new agents. In this study, despite the limitations of our testing paradigm, about 7% of the patients experienced clinical benefit from directed treatment encompassing multiple targetable pathways. These data support the utility of early mutational assessments and utilization of targeted therapies. As new agents that can exploit this mutational landscape find their way into clinical practice, the overall utility of this paradigm can only improve. Randomized controlled trials which are under way will shed more light on the subject.

## Conflicts of Interest

None.

## Supporting information


**Table S1**. Summary of previous treatments and genomic alterations among the 12 patients treated with genomic‐guided therapy.
